# Association of Statin Use With the In-Hospital Outcomes of 2019-Coronavirus Disease Patients: A Retrospective Study

**DOI:** 10.3389/fmed.2020.584870

**Published:** 2020-11-17

**Authors:** Yongzhen Fan, Tao Guo, Feifei Yan, Ming Gong, Xin A. Zhang, Chenze Li, Tao He, Huimin Luo, Lin Zhang, Ming Chen, Xiaoyan Wu, Hairong Wang, Ke-Qiong Deng, Jiao Bai, Lin Cai, Zhibing Lu

**Affiliations:** ^1^Department of Cardiology, Zhongnan Hospital of Wuhan University, Wuhan, China; ^2^Department of Orthopedics, Zhongnan Hospital of Wuhan University, Wuhan, China; ^3^Department of Medical Quality Control, Leishenshan Hospital, Wuhan, China; ^4^University of Oklahoma Health Science Center, Oklahoma City, OK, United States; ^5^Department of Ultrasonography, Zhongnan Hospital of Wuhan University, Wuhan, China

**Keywords:** COVID-19, statin, outcome, mortality, ARDS

## Abstract

**Background:** Statins have multiple protective effects on inflammation, immunity and coagulation, and may help alleviate pneumonia. However, there was no report focusing on the association of statin use with in-hospital outcomes of patients with coronavirus disease 2019 (COVID-19). We investigated the association between the use of statins and in-hospital outcomes of patients with COVID-19.

**Methods:** In this retrospective case series, consecutive COVID-19 patients admitted at 2 hospitals in Wuhan, China, from March 12, 2020 to April 14, 2020 were analyzed. A 1:1 matched cohort was created by propensity score-matched analysis. Demographic data, laboratory findings, comorbidities, treatments and in-hospital outcomes were collected and compared between COVID-19 patients taking and not taking statins.

**Result:** A total of 2,147 patients with COVID-19 were enrolled in this study. Of which, 250 patients were on statin therapy. The mortality was 2.4% (6/250) for patients taking statins while 3.7% (70/1,897) for those not taking statins. In the multivariate Cox model, after adjusting for age, gender, admitted hospital, comorbidities, in-hospital medications and blood lipids, the risk was lower for mortality (adjusted HR, 0.428; 95% CI, 0.169–0.907; *P* = 0.029), acute respiratory distress syndrome (ARDS) (adjusted HR, 0.371; 95% CI, 0.180–0.772; *P* = 0.008) or intensive care unit (ICU) care (adjusted HR, 0.319; 95% CI, 0.270–0.945; *P* = 0.032) in the statin group vs. the non-statin group. After propensity score-matched analysis based on 18 potential confounders, a 1:1 matched cohort (206:206) was created. In the matched cohort, the Kaplan-Meier survival curves showed that the use of statins was associated with better survival (*P* = 0.025). In a Cox regression model, the use of statins was associated with lower risk of mortality (unadjusted HR, 0.254; 95% CI, 0.070–0.926; *P* = 0.038), development of ARDS (unadjusted HR, 0.240; 95% CI, 0.087–0.657; *P* = 0.006), and admission of ICU (unadjusted HR, 0.349; 95% CI, 0.150–0.813; *P* = 0.015). The results remained consistent when being adjusted for age, gender, total cholesterol, triglyceride, low density lipoprotein cholesterol, procalcitonin, and brain natriuretic peptide. The favorable outcomes in statin users remained statistically significant in the first sensitivity analysis with comorbid diabetes being excluded in matching and in the second sensitivity analysis with chronic obstructive pulmonary disease being added in matching.

**Conclusion:** In this retrospective analysis, the use of statins in COVID-19 patients was associated with better clinical outcomes and is recommended to be continued in patients with COVID-19.

## Introduction

Coronavirus disease 2019 (COVID-19) has already been a global pandemic since early December 2019. Since then, infection with COVID-19 has rapidly spread throughout the world, even causing widespread social and economic disruption (1, 2). At the time of submission, the total number of infected patients has risen to 43, 251, 698 around the world, with 1,154,214 associated deaths. However, thus far, there are no specific therapies or vaccines available for COVID-19. Most of the currently used clinical interventions are symptomatic supportive therapies, which have exhibited limited therapeutic effects for COVID-19.

It is worth noticing that patients with common comorbidities, including hypertension and cardiovascular diseases are at greater risk for severe acute respiratory syndrome coronavirus 2 (SARS-CoV-2) infection and its related acute respiratory distress syndrome (ARDS) and mortality (3). Most of these patients are taking statins routinely based on cardiovascular guidelines. Statins, as one of the inhibitors of 3-hydroxy-3-methylglutaryl coenzyme A reductase (HMG-CoA), are a class of lipid-lowering medications and are frequently used in patients with cardiovascular diseases or to prevent cardiovascular events (4–6). Statins are also well-known for their potential immunomodulatory and anti-inflammatory effects in pneumonia (7, 8). An earlier retrospective cohort study showed that, in bacterial pneumonia patients, in-hospital mortality was significantly reduced after using statins (9). Many researchers have focused on statins in the treatment of infections (10–12). In 2014, some researchers suggested that statins might be used for treatment of patients with Ebola virus disease (13). Although most of these studies argue that statins are advantageous to outcomes and prognosis of patients with pneumonia, Fernandez et al. demonstrated that hospital mortality was significantly higher after statin therapy (14). Therefore, whether statin use was associated with reduced mortality for patients with pneumonia is still in debate. To the best of our knowledge, there is no clinical or experimental data focusing on the effects of statin use on the in-hospital outcomes of COVID-19 patients. The main purpose of the present study was to investigate the association of the statin use with the in-hospital outcomes of COVID-19 patients.

## Methods

### Study Design and Participants

This retrospective study was performed at Zhongnan Hospital of Wuhan University and Leishenshan Hospital in Wuhan, China, which were designated hospitals to treat patients with COVID-19. Leishenshan Hospital was taken over by Zhongnan Hospital of Wuhan University during the epidemic period. The inclusion criteria included patients with COVID-19 who were admitted to the 2 hospitals from March 12, 2020 to April 14, 2020 and who were either discharged with following recovery or died during hospitalization. The exclusion criteria included incomplete medical records, loss of follow-up due to being transferred to other hospitals and discontinuation of use of statins. COVID-19 was diagnosed according to the interim guidance of the World Health Organization (15). The treatment strategies for COVID-19 in the 2 hospitals were based on Chinese Guideline of Clinical Management for COVID-19 (1st−7th version). Patients with critical illness (severe respiratory failure, shock, and multiple organ dysfunction) were transferred to the intensive care unit (ICU). This study complied with the edicts of the 1975 Declaration of Helsinki (16) and was approved by the institutional ethics board of Zhongnan Hospital of Wuhan University (No. 2020026). The patients' consents were obtained from individual participant or their relatives.

### Data Collection

The electronic medical records of the patients with confirmed SARS-CoV-2 infection by real-time reverse-transcription PCR (RT-PCR) were extracted and reviewed by a trained team of physicians from the 2 hospitals during the epidemic period. The data including demographics, medical history, laboratory examinations, comorbidities, complications, treatments, and outcomes were collected and analyzed. The researchers were responsible to contact the patients or their families in case of uncertainties about the data to ensure to maximum the accuracy of our data.

### In-hospital Outcomes

The main in-hospital outcomes included COVID-19 related death or discharge. Successful treatment toward hospital cure for the patients with COVID-19 comprised all of the following criteria: relieved clinical symptoms, normal body temperature, significant resolution of inflammation as shown by chest radiography and at least 2 consecutive negative results assessed by RT-PCR assay for SARS-Cov-2 (17). The secondary outcomes included development of ARDS and requirement of ICU care. The definition of ARDS required bilateral infiltrates on chest radiograph consistent with pulmonary edema and partial pressure of arterial oxygen/fraction of inspired oxygen <300 mmHg.

### Propensity Score-Matched (PSM) Analysis and Sensitivity Analysis

To validate the findings, propensity score-matched (PSM) cohort was created based on 18 baseline variables which were expected to be potential confounders. Statin and non-statin users were paired according to the propensity scores using nearest matching with a caliper size of 0.05. The balance of covariates was evaluated by estimating standardized differences (SD) and *p*-values before and after matching, and SD absolute value <0.1 was considered perfect balancing between the two groups. The imbalanced variables with SD ≥ 0.1 in PSM analysis were adjusted in the following multivariate Cox model.

We performed two sensitivity analyses to evaluate the robustness of propensity score-matched cohort analyses. In the first sensitivity analysis, comorbid diabetes was not excluded in matching. In the second sensitivity analysis, chronic obstructive pulmonary disease (COPD) was added in matching.

### Statistical Analysis

Categorical variables are shown as frequencies and percentages, and continuous variables as mean ± standard deviation, or median (interquartile range). The means for continuous variables were compared using independent group *t*-tests when the data were normally distributed, otherwise, the Mann–Whitney *U*-test was used. Proportions for categorical variables were compared using the *X*^2^-test, although Fisher's exact test was used when data were limited. A Kaplan–Meier plot was used for survival data. We compared the in-hospital outcomes of patients who did and did not use statins by using Cox proportional hazards models to calculate hazard ratios and 95% confidence intervals. In the unmatched cohort, the variables which were considered to confound the association of statin use with the clinical outcome were adjusted for in the Cox regression model. Additionally, we adjusted for imbalanced variables with SD ≥ 0.1 in PSM analysis in following multivariate Cox model. All statistical analyses were performed with SPSS19.0 for Windows. A two-tailed *P* < 0.05 was considered statistically significant.

## Results

### Demographics Data

**Figure 1:**
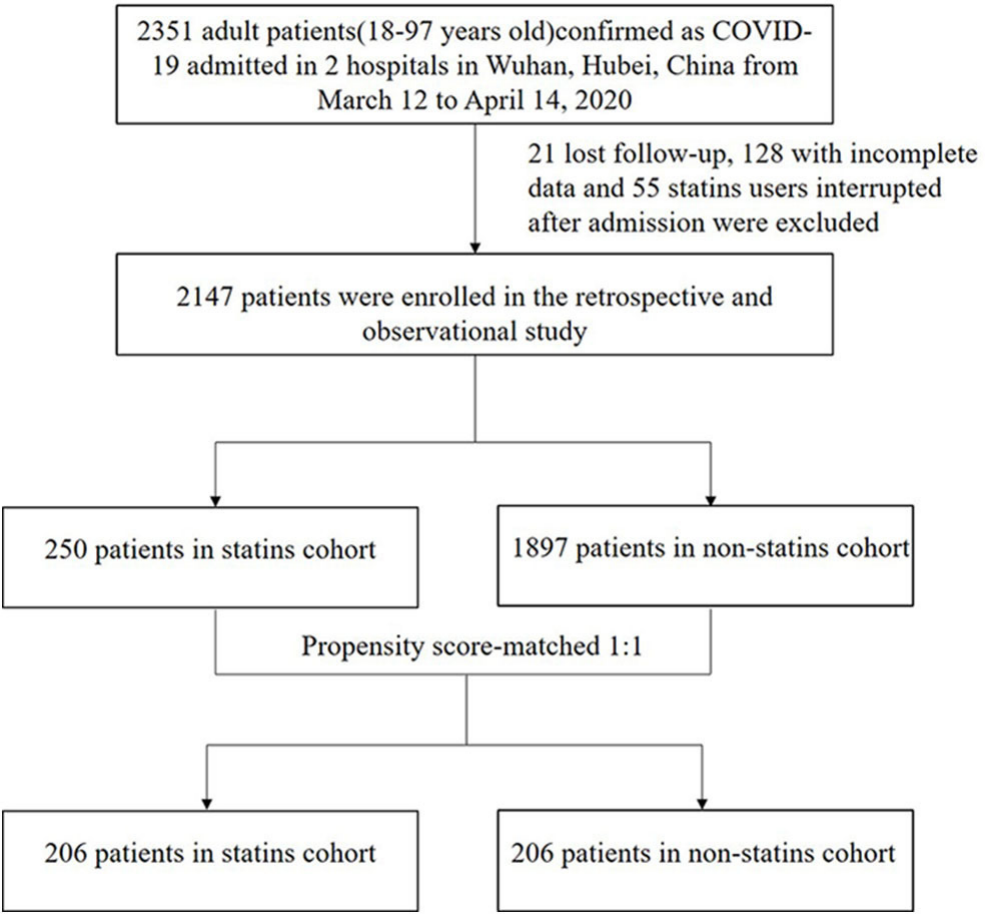
The flowchart showing the strategy of participant enrollment COVID-19, coronavirus disease 2019.

A total of 2,351 consecutive patients hospitalized with COVID-19 who were successfully treated and discharged or died at Zhongnan Hospital of Wuhan University or Leishenshan Hospital from March 12 to April 14, 2020 were analyzed (Figure 1). By reviewing all electronic medical records, 128 patients with incomplete data, 55 patients who were on statins prior to admission but interrupted after admission and 21 patients who had been transferred to other hospitals and lost follow-up were excluded. Finally, 2,147 patients with COVID-19 were enrolled in this study. Of which, 250 patients were on statins prior to admission and continued their use during hospitalization including 162 (64.8%) atorvastatin (20 mg every day) and 75 (30.0%) rosuvastatin (10 mg every day), and the remaining 13 (5.2%) patients used other statins such as pravastatin. The remaining 1,897 patients never used statins.

### Clinical Characteristics on Admission Before and After PSM Analysis

The comorbid coronary heart disease, hypertension, diabetes, cerebrovascular diseases were more frequent in the statin users than the non-statin users (Table 1). Many laboratory results including neutrophil count, prothrombin time, activated partial thromboplastin time, D-dimer, total cholesterol (TC), triglyceride, low density lipoprotein cholesterol (LDL-C), procalcitonin, creatine kinase-MB, high-sensitivity troponin I (hs-TnI), and brain natriuretic peptide (BNP) showed significant (*p* < 0.05) or marginal significant (*p* < 0.10) differences between the two groups on admission (Table 2). Since many significantly imbalanced variables existed in the baseline state on admission, the outcomes could not be directly compared between the two groups. Then the PSM analysis was conducted to account for these 18 potential confounding factors (Supplementary Table 1). A new cohort of patients were matched in the statin group *vs*. non-statin group at a ratio of 1:1 with 206 patients in each group. In the matched cohort, all the variables in Table 1 did not show significant differences (all *p* > 0.05), however, the variables with SD > 0.1 including TC, triglyceride, LDL-C, procalcitonin, and BNP did not achieve a perfect matching, therefore, along with age and sex, these variables entered in the multivariate COX model for adjustment (Table 3). One hundred and forty-four (69.9%) patients were on atorvastatin (20 mg every day), 61 (29.6%) were on rosuvastatin (10 mg every day) and the other one patient was on pravastatin. The treatments were comparable in the unmatched and matched cohorts (Table 1).

**Table 1:** Demographics, clinical characteristics, and treatments of COVID-19 patients.

	**Unmatched**			**Matched**		
	**Non-statin**	**Statin**	***P*-value**	**Non-Statin**	**Statin**	***P*-value**
Number of patients	1,897	250		206	206	
Male-counts (%)	926 (48.8)	115 (46.0)	0.403	80 (38.8)	90 (43.7)	0.317
Age-years	58 (48, 68)	66 (57, 72)	<0.001	66 (57, 73)	64 (57, 72)	0.556
Smoker-counts (%)	133 (7.0)	21 (8.4)	0.423	18 (8.7)	19 (9.2)	0.863
Heart Rate, bpm	83 (75, 92)	85 (77, 99)	0.661	84 (76, 94)	85 (75, 98)	0.780
SBP, mmHg	130 (124, 140)	133 (122, 145)	0.311	132 (124, 141)	133 (123, 145)	0.564
DBP, mmHg	81 (73, 95)	83 (73, 98)	0.552	82 (72, 97)	83 (73, 97)	0.785
Fever-counts (%)	1,332 (70.2)	167 (66.8)	0.269	144 (69.9)	139 (67.5)	0.595
Hospitalization-days^a^	17 (12, 24)	16 (12, 21)	0.589	16 (12, 23)	16 (16, 20)	0.799
**Comorbidities-count (%)**						
Hypertension	575 (30.3)	130 (52.0)	<0.001	106 (51.5)	102 (49.5)	0.693
Coronary heart disease	97 (5.1)	68 (27.2)	<0.001	44 (21.4)	41 (19.9)	0.715
Diabetes	239 (12.6)	54 (21.6)	<0.001	41 (19.9)	41 (19.9)	>0.999
Cerebrovascular diseases	43 (2.3)	27 (10.8)	<0.001	13 (6.3)	16 (7.8)	0.563
COPD	35 (1.8)	4 (1.6)	>0.999	10 (4.9)	3 (1.5)	0.087
Chronic hepatic dysfunction	55 (2.9)	9 (3.6)	0.540	10 (4.9)	9 (4.4)	0.814
Chronic renal dysfunction	63 (3.3)	10 (4.0)	0.578	10 (4.9)	7 (3.4)	0.457
Malignancy	45 (2.4)	4 (1.6)	0.651	11 (5.3)	4 (1.9)	0.112
**Treatments-count (%)**						
Antiviral therapy	1,726 (90.9)	230 (92.0)	0.596	181 (87.9)	187 (90.7)	0.543
Antibiotic therapy	813 (42.9)	110 (44.0)	0.732	91 (44.2)	91 (44.2)	>0.999
Glucocorticoid therapy	173 (9.1)	24 (9.6)	0.805	21 (10.2)	16 (7.8)	0.389
Immunoglobulin therapy	61 (3.2)	7 (2.8)	0.724	4 (1.9)	5 (2.4)	0.751
ACEI/ARB	106 (5.6)	38 (15.2)	<0.001	22 (10.7)	33 (16.0)	0.111
Mechanical ventilation	180 (9.4)	26 (10.4)	0.646	22 (10.6)	17 (8.2)	0.400
CRRT	47 (2.5)	3 (1.2)	0.267	6 (2.9)	2 (1.0)	0.284
ECMO	9 (0.5)	0 (0)	0.610	4 (1.9)	0 (0)	0.123

*Values are median (interquartile range), mean ± standard deviation or n (%)*.

a *Hospitalization indicates days from admission to death/discharge. COVID-19, coronavirus disease 2019; ACEI, angiotensin-converting enzyme inhibitor; ARB, angiotensin receptor blocker; SBP, systolic blood pressure; DBP, diastolic blood pressure; COPD, chronic obstructive pulmonary disease; CRRT, continuous renal replacement therapy; ECOM, extracorporeal membrane oxygenation*.

**Table 2:** Laboratory results among different groups on admission.

	**Unmatched**			**Matched**		
	**Non-statin**	**Statin**	***P*-value**	**Non-statin**	**Statin**	***P*-value**
Number of patients, *n*	1,897	250		206	206	
White blood cell count, ×10^9^/L	5.21 (3.58, 6.64)	5.41 (3.94, 6.93)	0.203	5.21 (3.40, 6.65)	5.27 (3.89, 6.82)	0.530
Neutrophil count, ×10^9^/L	3.28 (2.53, 4.39)	3.49 (2.60, 4.72)	0.049	3.29 (2.48, 4.21)	3.43 (2.59, 4.65)	0.218
Lymphocyte count, ×10^9^/L	1.54 (1.16, 1.93)	1.59 (1.10, 1.96)	0.610	1.46 ± 0.61	1.60 ± 0.66	0.028
Hemoglobin, g/L	126 (114, 136)	123 (114, 135)	0.214	122 (110, 130)	124 (115, 135)	0.016
Platelet, ×10^9^/L	225 (182, 274)	224 (182, 290)	0.608	216 ± 74	239 ± 78	0.002
D-dimer, μg/mL	0.45 (0.23, 1.26)	0.62 (0.30, 1.58)	0.001	0.47 (0.31, 1.28)	0.47 (0.32, 1.10)	0.767
Total cholesterol, mmol/L	4.23 (3.62, 4.81)	4.16 (3.45, 5.36)	0.455	4.15 (3.48, 4.98)	4.22 (3.50, 5.15)	0.386
Triglyceride, mmol/L	1.17 (0.79, 1.70)	1.39 (0.87, 2.10)	<0.001	0.68 (0.45, 1.00)	1.37 (0.89, 1.91)	<0.001
HDL-C, mmol/L	1.13 (0.94, 1.36)	1.13 (0.94, 1.32)	0.910	1.30 ± 0.40	1.17 ± 0.29	<0.001
LDL-C mmol/L	2.54 ± 0.70	2.61 ± 0.95	0.284	2.41 (1.87, 2.92)	2.50 (1.98, 3.11)	0.131
Serum potassium, mmol/L	4.33 (4.03, 4.62)	4.34 (4.00, 4.67)	0.510	4.34 ± 0.50	4.35 ± 0.54	0.888
Serum calcium, mmol/L	2.18 (2.10, 2.24)	2.18 (2.09, 2.25)	0.637	2.18 (2.10, 2.25)	2.15 (2.07, 2.21)	0.001
CRP, mg/L	1.62 (0.53, 6.11)	1.50 (0.53, 7.20)	0.834	2.28 (0.65, 13.7)	1.50 (0.54, 6.14)	0.042
Procalcitonin, ng/mL	0.04 (0.03, 0.06)	0.04 (0.03, 0.07)	0.042	0.04 (0.03, 0.06)	0.04 (0.03, 0.06)	0.425
Creatine kinase, U/L	53.0 (36.8, 79.0)	55.0 (35.0, 77.8)	0.185	54.5 (39.0, 83.1)	57.4 (35.0, 87.8)	0.091
Creatine kinase–MB, ng/mL	1.16 (0.80, 1.94)	1.36 (0.98, 2.04)	0.012	1.19 (1.19,1.21)	1.19 (1.19,1.49)	0.256
hs-TnI, ng/mL	0.01 (0.010, 0.011)	0.01 (0.010, 0.013)	0.084	0.01 (0.010, 0.013)	0.01 (0.010, 0.013)	0.261
BNP, pg/mL	0.01 (0.01, 44.6)	14.4 (0.010, 83.2)	0.025	10 (10.0, 21.9)	10 (10.0, 22.2)	0.792
Alanine aminotransferase,U/L	23.0 (15.0, 38.0)	22.1 (15.0, 38.0)	0.781	19.0 (12.3, 34.9)	21.9 (15.0, 36.0)	0.026
Aspartate aminotransferase,U/L	20.0 (16.0, 28.0)	20.0 (16.0, 28.0)	0.766	20.0 (16.0, 28.8)	20.0 (16.0, 26.7)	0.376
Creatinine, μmol/L	64.6 (54.2, 77.2)	64.9 (55.5, 78.3)	0.496	63.9 (56.4, 77.7)	63.9 (55.6, 77.9)	0.864
PaO_2_/FiO_2_, mmHg	351 (312, 387)	335 (303, 372)	0.113	366 (318, 389)	335 (309, 370)	0.075

*Values are median (interquartile range), mean ± standard deviation. HDL-C, high density lipoprotein cholesterol; LDL-C, low density lipoprotein cholesterol; CRP, C-reactive protein; hs-TnI, high-sensitivity troponin I; BNP, brain natriuretic peptide; PaO2/FiO2, partial pressure of arterial oxygen to the fraction of inspired oxygen*.

**Table 3:** Hazard ratio for In-hospital outcomes of COVID-19 patients in matched cohort.

	**Total**	**Non-statin**	**Statin**	***P*-value**	**HR**	**95% CI**
**Unadjusted outcomes-count (%)**						
Death-count	13 (3.2)	10 (4.9)	3 (1.5)	0.038	0.254	0.070–0.926
ARDS	22 (5.3)	16 (7.8)	6 (2.9)	0.006	0.240	0.087–0.657
ICU admission	26 (6.3)	17 (8.3)	9 (4.4)	0.015	0.349	0.150–0.813
**Adjusted outcomes-count (%)**						
Death-count	13 (3.2)	10 (4.9)	3 (1.5)	0.037	0.251	0.068–0.923
ARDS	22 (5.3)	16 (7.8)	6 (2.9)	0.034	0.232	0.060–0.894
ICU admission	26 (6.3)	17 (8.3)	9 (4.4)	0.031	0.381	0.158–0.915

*Values are n (%). P-value was acquired by using the COX model. COVID-19, coronavirus disease 2019; ARDS, acute respiratory distress syndrome; ICU, intensive care unit; HR, hazard ratio; CI, confidence interval*.

*Adjusted variables included age, gender, TC, triglyceride, LDL-C, procalcitonin, and BNP*.

### In-hospital Outcomes

In the unmatched cohort, the mortality is 2.4% (6/250) for patients taking statins while 3.7% (70/1,897) for those not taking statins. In the multivariate Cox model, after adjusting for age, gender, admitted hospital, comorbidities (hypertension, coronary heart disease, diabetes, cerebrovascular diseases), in-hospital medications (ACEI/ARB, glucocorticoid) and blood lipids (TC, LDL-C), statin use was associated with lower mortality (adjusted HR, 0.428; 95% CI, 0.169–0.907; *P* = 0.029), ARDS (adjusted HR, 0.371; 95% CI, 0.180–0.772; *P* = 0.008) or ICU care (adjusted HR, 0.319; 95% CI, 0.270–0.945; *P* = 0.032) vs. non-statin use.

**Figure 2:**
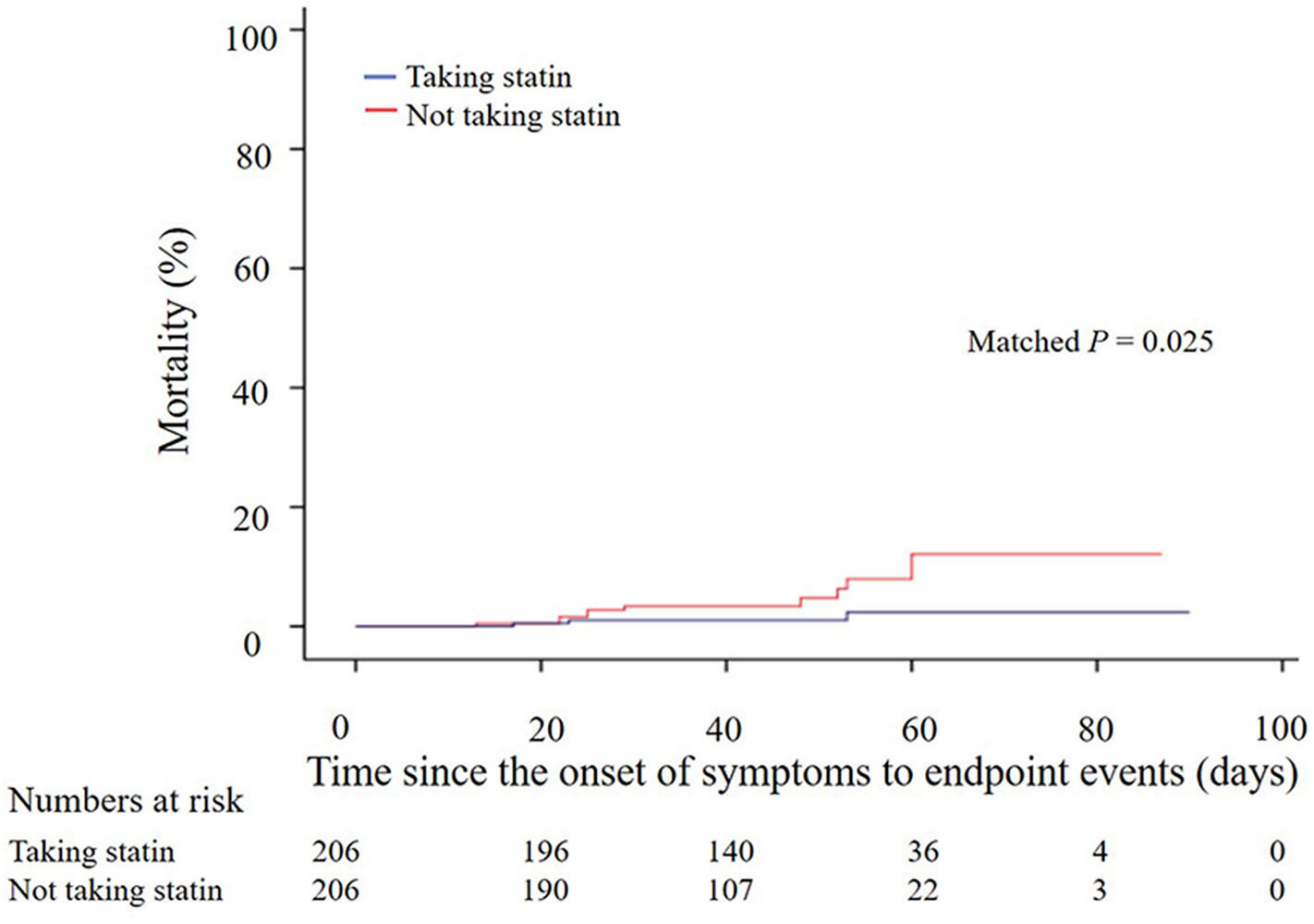
Kaplan–Meier survival curves of 412 COVID-19 patients with and without taking statins (206:206) after propensity score-matched analysis. COVID-19, coronavirus disease 2019.

In the matched cohort, as shown in the Kaplan-Meier survival curves, the use of statins was associated with better survival (*P* = 0.025; Figure 2). In a Cox regression model, the use of statins was associated with lower risk of in-hospital mortality (unadjusted HR, 0.254; 95% CI, 0.070–0.926; *P* = 0.038), development of ARDS (unadjusted HR, 0.240; 95% CI, 0.087–0.657; *P* = 0.006), and admission of ICU (unadjusted HR, 0.349; 95% CI, 0.150–0.813; *P* = 0.015). The results remained consistent when being adjusted for age, gender, TC, triglyceride, LDL-C, procalcitonin, and BNP (Table 3).

### Sensitivity Analyses

Additional sensitivity analyses aiming to further assess the robustness of the association between statin use and outcomes were performed, the results remained consistent and statistically significant in the first sensitivity analysis with comorbid diabetes being excluded in matching and in the second sensitivity analysis with COPD being added in matching (Supplementary Table 2).

## Discussion

### Main Findings

The present study demonstrated for the first time that, the continuous use of statins was associated with lower mortality, less development of ARDS and less requirement of ICU care. Even after matched or adjusted for the blood lipids, the results remained consistently significant, indicating that this association was independent of its lipid-lowering effect.

### Association of the Statin Use With the In-hospital Outcomes for Patients With COVID-19

The observations regarding the association of statin use with the outcomes of types of pneumonia had been reported previously (18, 19). Overall, significant decrease in mortality was demonstrated in the hospitalized pneumonia patients taking statins. A previous retrospective cohort study showed that statin use was associated with a decreased risk of mortality in patients hospitalized with community-acquired pneumonia (20). In another study, continuous use of statins was also correlated with a decreased risk of mortality or intubation in patients with COPD (21) Consistent with above observations, we found a decrease in mortality, less development of ARDS, and less requirement of ICU care in patients with continuous use of statins during their hospitalization for COVID-19.

The current study further showed some laboratory indicators reflecting inflammation (C-reactive protein, procalcitonin) and myocardial injury (hs-TNI, BNP) on admission could not be perfectly matched by PSM analysis. They might be still resistant confounders which had been adjusted in the following multivariate Cox model. However, there is another possibility that, they might be mediators. Due to routine use of statins before admission, the pharmacological actions of statins on COVID-19 patients may be already significant since the patients were initially infected with COVID-19. This postulation might be supported by the findings of elevated alanine transaminase and creatine kinase and decreased calcium concentration on admission, which were frequently-observed side effects in long-term use of statins, especially in those with complications of liver injury and rhabdomyolysis.

### Possible Mechanisms for the Association Between Statin Use and Favorable Outcomes of COVID-19

The potential benefits of statins for patients with COVID-19 are presumably related to their multiple effects. In addition to their benefits in patients with cardiovascular or cerebrovascular diseases, the pleiotropic effects including anti-inflammatory, anti-thrombotic, immunomodulatory, and reducing reactive oxygen species have also been reported (22). The evidence for the anti-inflammatory properties of statins in the lung and their potential role as novel treatments for respiratory diseases have been noticed (23). Our findings were consistent with these reports and demonstrated that the use of statins was associated with less development of ARDS. This association was beyond its lipid-lowering effect.

Increased level of angiotensin II was observed in COVID-19-affected individuals, which was demonstrated to be correlated with viral load and severity of illness of COVID-19 (24). As the isoenzyme of angiotensin converting enzyme (ACE), ACE2 plays a protective role in generating angiotensin-(1-7) (Ang 1-7) from angiotensin II in renin-angiotensin-aldosterone system (RAAS). When patients are infected with SARS-CoV-2, their ACE2 is further down-regulated by binding it with SARS-CoV-2 resulting in a deterioration of the imbalance of ACE2/ACE, and subsequently induces a sharp release of AngII by over-activation of RAAS. The upregulation of ACE2 by the use of statins was reported previously (25, 26), and may be another important mechanism of statin benefits for COVID-19. However, the exact mechanisms underlying the association between statin use and in-hospital outcomes need to be validated by further experimental studies and clinical observations.

### Comparisons to Other Studies

Statins are well-known for their anti-inflammatory effects, and some hospitals included them in the COVID-19 treatment protocol (27). However, whether statin use was associated with reduced mortality for patients with COVID-19 is still in debate. Especially, some scholars have an opinion that statins should be used with caution in COVID-19 patients because it could cause myalgia, myopathies, or rhabdomyolysis, thereby exacerbating the disease (28), yet it should be noted that both of statin induced myopathies and the deterioration of SARSCoV-2 infection are characterized as elder, and liver and kidney dysfunction. Thus, it is hard to differentiate the appearance of myalgia, rhabdomyolysis, increased creatine phosphokinase, and acute kidney injury in COVID-19 from statin therapy (29). Furthermore, there were other studies showed that COVID-19 patients could not benefit from the administration of statin (30–32). This discrepancy may result from the limited sample size and the heterogeneity of study population. Such as in the study from Dreher et al. only 50 patients with COVID-19 were included (31), and in the case series of 1,000 COVID-19 patients, they included all mild-to-critical patients with tested positive for COVID-19 (32). Nevertheless, a retrospective analysis of 154 COVID-19 patients reported that the use of statin could significantly reduce the severity of COVID-19 among nursing home residents (33), which is consistent with our findings. Overall, based on our results, continuation of statin therapy among COVID-19 patients with a history of atherosclerotic cardiovascular disease or diabetes was recommended.

### Study Limitations

Our study has several limitations. First, with all the limitations of a retrospective study and relatively small sample size, further randomized prospective studies with more patients are required to verify the findings in our study. However, the propensity score-matched analysis, sensitivity analyses, and multivariate Cox model have been further conducted to limit potential bias or exclude possible confounders. The results from these analyses showed consistent findings and strengthened our conclusion. Even so, a cause-and-effect relationship between statins and survival cannot be inferred and only an association between statins and favorable outcomes was reported in the present study. Second, some specific information regarding cardiovascular complications and inflammation such as echocardiography and interleukin-6 were not included in the study due to the limited conditions in the isolation ward and the urgency of constraining the COVID-19 epidemic.

### Conclusion

The continuous use of statins was associated with favorable outcomes in patients with COVID-19. The statin use is recommended to be continued in patients with COVID-19. However, given that this study was a retrospective analysis, further prospective studies and randomized clinical trials are warranted to verify the beneficial effect of statin in COVID-19 patients and in its different subgroups.

## Data Availability Statement

The original contributions presented in the study are included in the article/Supplementary Material, further inquiries can be directed to the corresponding author/s.

## Ethics Statement

This study was approved by the Institutional Ethics Board of Zhongnan Hospital of Wuhan University (No. 2020026). The patients' consents were obtained from individual participant or their relatives.

## Author Contributions

All authors listed have made a substantial, direct and intellectual contribution to the work, and approved it for publication.

## Conflict of Interest

The authors declare that the research was conducted in the absence of any commercial or financial relationships that could be construed as a potential conflict of interest.

## References

[1] Huang C Wang Y Li X Ren L Zhao J Hu Y. Clinical features of patients infected with 2019 novel coronavirus in Wuhan, China. Lancet. (2020) 395:497–506. 10.1016/S0140-6736(20)30183-5 31986264 PMC7159299

[2] Holshue ML DeBolt C Lindquist S Lofy KH Wiesman J Bruce H First case of 2019 novel coronavirus in the United States. N Engl J Med. (2020) 382:929–36. 10.1056/NEJMoa2001191 32004427 PMC7092802

[3] Wu C Chen X Cai Y Zhou X Xu S Huang H. Risk factors associated with acute respiratory distress syndrome and death in patients with coronavirus disease 2019 pneumonia in Wuhan, China. JAMA Intern Med. (2020) 180:934–43. 10.1001/jamainternmed.2020.0994 32167524 PMC7070509

[4] Viasus D Garcia-Vidal C Gudiol F Carratalà J. Statins for community-acquired pneumonia: current state of the science. Eur J Clin Microbiol Infect Dis. (2010) 29:143–52. 10.1007/s10096-009-0835-0 19943074

[5] Schuster H Barter PJ Stender S Cheung RC Bonnet J Morrell JM. Effects of switching statins on achievement of lipid goals: measuring effective reductions in cholesterol using rosuvastatin therapy (MERCURY I) study. Am Heart J. (2004) 147:705–13. 10.1016/j.ahj.2003.10.004 15077101

[6] Nissen SE Tuzcu EM Schoenhagen P Crowe T Sasiela WJ Tsai J. Statin therapy, LDL cholesterol, C-reactive protein, and coronary artery disease. N Engl J Med. (2005) 352:29–38. 10.1056/NEJMoa042000 15635110

[7] Gowdy KM Fessler MB. Emerging roles for cholesterol and lipoproteins in lung disease. Pulm Pharmacol Ther. (2013) 26:430–7. 10.1016/j.pupt.2012.06.002 22706330 PMC3466369

[8] Chalmers JD Short PM Mandal P Akram AR Hill AT. Statins in community acquired pneumonia: evidence from experimental and clinical studies. Respir Med. (2010) 104:1081–91. 10.1016/j.rmed.2010.04.005 20447815

[9] Liappis AP Kan VL Rochester CG Simon GL. The effect of statins on mortality in patients with bacteremia. Clin Infect Dis. (2001) 33:1352–7. 10.1086/323334 11565076

[10] Björkhem-Bergman L Bergman P Andersson J Lindh JD. Statin treatment and mortality in bacterial infections–a systematic review and meta-analysis. PloS ONE. (2010) 5:e10702. 10.1371/journal.pone.0010702 20502712 PMC2873291

[11] Janda S Young A Fitzgerald JM Etminan M Swiston J. The effect of statins on mortality from severe infections and sepsis: a systematic review and meta-analysis. J Crit Care. (2010) 25:656.e7–22. 10.1016/j.jcrc.2010.02.013 20413251

[12] Tleyjeh IM Kashour T Hakim FA Zimmerman VA Erwin PJ Sutton AJ. Statins for the prevention and treatment of infections: a systematic review and meta-analysis. Arch Intern Med. (2009) 169:1658–67. 10.1001/archinternmed.2009.286 19822822

[13] Fedson DS. A practical treatment for patients with Ebola virus disease. J Infect Dis. (2015) 211:661–2. 10.1093/infdis/jiu474 25160984

[14] Fernandez R De Pedro VJ Artigas A Statin therapy prior to ICU admission: protection against infection or a severity marker? Intens Care Med. (2006) 32:160–4. 10.1007/s00134-005-2743-9 16086178

[15] World Health Organization Clinical Management of Severe Acute Respiratory Infection When Novel Coronavirus (nCoV) Infection is Suspected: Interim Guidance. WHO (2020).

[16] World Medical Association World medical association declaration of helsinki: ethical principles for medical research involving human subjects. JAMA. (2013) 310:2191–4. 10.1001/jama.2013.281053 24141714

[17] China NHCotPsRo. Chinese Management Guideline for COVID-19 (Version 7.0). (2020).32553908

[18] Chopra V Rogers MA Buist M Govindan S Lindenauer PK Saint S. Is statin use associated with reduced mortality after pneumonia? A systematic review and meta-analysis. Am J Med. (2012) 125:1111–23. 10.1016/j.amjmed.2012.04.011 22835463

[19] Henry C Zaizafoun M Stock E Ghamande S Arroliga AC White HD. Impact of angiotensin-converting enzyme inhibitors and statins on viral pneumonia. Proc. (2018) 31:419–23. 10.1080/08998280.2018.1499293 30948970 PMC6414001

[20] Mortensen EM Pugh MJ Copeland LA Restrepo MI Cornell JE Anzueto A. Impact of statins and angiotensin-converting enzyme inhibitors on mortality of subjects hospitalised with pneumonia. Eur Respir J. (2008) 31:611–7. 10.1183/09031936.00162006 17959631

[21] Blamoun AI Batty GN DeBari VA Rashid AO Sheikh M Khan MA. Statins may reduce episodes of exacerbation and the requirement for intubation in patients with COPD: evidence from a retrospective cohort study. Int J Clin Pract. (2008) 62:1373–8. 10.1111/j.1742-1241.2008.01731.x 18422598

[22] Crea F Libby P. Acute coronary syndromes: the way forward from mechanisms to precision treatment. Circulation. (2017) 136:1155–66. 10.1161/CIRCULATIONAHA.117.029870 28923905 PMC5679086

[23] Hothersall E McSharry C Thomson NC. Potential therapeutic role for statins in respiratory disease. Thorax. (2006) 61:729–34. 10.1136/thx.2005.057976 16877692 PMC2104700

[24] Liu Y Yang Y Zhang C Huang F Wang F Yuan J. Clinical and biochemical indexes from 2019-nCoV infected patients linked to viral loads and lung injury. Sci China Life Sci. (2020) 63:364–74. 10.1007/s11427-020-1643-8 32048163 PMC7088566

[25] Wosten-van Asperen RM Lutter R Specht PA Moll GN van Woensel JB van der Loos CM Acute respiratory distress syndrome leads to reduced ratio of ACE/ACE2 activities and is prevented by angiotensin-(1-7) or an angiotensin II receptor antagonist. J Pathol. (2011) 225:618–27. 10.1002/path.2987 22009550

[26] Tikoo K Patel G Kumar S Karpe PA Sanghavi M Malek V. Tissue specific up regulation of ACE2 in rabbit model of atherosclerosis by atorvastatin: role of epigenetic histone modifications. Biochem Pharmacol. (2015) 93:343–51. 10.1016/j.bcp.2014.11.013 25482567

[27] Massachusetts General Hospital Massachusetts General Hospital COVID-19 Treatment Guidance Version 1.0. (2020). Available online at: https://medtube.net/infectiousdiseases/medical-documents/26086-covid19-treatmentguidelines-by-massachusetts-general-hospital (accessed March 28, 2020).

[28] Subir R Jagat JM Kalyan KG Pros and cons for use of statins in people with coronavirus disease-19 (COVID-19). Diabetes Metab Syndr. (2020) 14:1225–9. 10.1016/j.dsx.2020.07.011 32683320 PMC7352102

[29] Karalis DG. Are statins safe in patients with COVID-19? J Clin Lipidol. (2020) 14:396–7. 10.1016/j.jacl.2020.06.009 32682805 PMC7308024

[30] Hariyanto TI Kurniawan A. Statin therapy did not improve the in-hospital outcome of coronavirus disease 2019 (COVID-19) infection. Diabetes Metab Syndr. (2020) 14:1613–5. 10.1016/j.dsx.2020.08.023 32882643 PMC7448951

[31] Dreher M Kersten A Bickenbach J Balfanz P Hartmann B Cornelissen C. The characteristics of 50 hospitalized COVID-19 patients with and without ARDS. Dtsch Arztebl Int. (2020) 117:271–8. 10.3238/arztebl.2020.0271 32519944 PMC7171478

[32] Argenziano MG Bruce SL Slater CL Tiao JR Baldwin MR Barr RG. Characterization and clinical course of 1000 patients with coronavirus disease 2019 in New York: retrospective case series. BMJ. (2020) 369:m1996. 10.1136/bmj.m1996 32471884 PMC7256651

[33] De Spiegeleer A Bronselaer A Teo JT Byttebier G De Tre G Belmans L. The effects of ARBs, ACEis, and statins on clinical outcomes of COVID-19 infection among nursing home residents. J Am Med Dir Assoc. (2020) 21:909–14.e902. 10.1016/j.jamda.2020.06.018 32674818 PMC7294267

