# Supplementary material for: Association of Statin Use With the In-Hospital Outcomes of 2019-Coronavirus Disease Patients: A Retrospective Study
Source: Front Med (Lausanne). 2020 Nov 17;7:584870. doi: 10.3389/fmed.2020.584870 (PMC7717990; doi:10.3389/fmed.2020.584870)
Supplement: Supplementary file 1 [file Table_1.docx]

**Supplement table 1** Propensity score matching analysis.

|  |  | Unmatched |  |  |  | Matched |  |
| --- | --- | --- | --- | --- | --- | --- | --- |
|  | Non-Statin | Statin | SD |  | Non-Statin | Statin | SD |
| Number of patients | 1897 | 250 |  |  | 206 | 206 |  |
| Hospital^a^ -count (%) | 1771/126 | 235/15 | 0.106 |  | 199/7 | 197/9 | 0.050 |
| Male-counts (%) | 926(48.8) | 115(46.0) | 0.160 |  | 80(38.8) | 90(43.7) | 0.098 |
| Age-years | 58(48, 68) | 65.5(57, 72) | 0.142 |  | 66(57, 73) | 64(57, 72) | 0.034 |
| Hypertension | 575(30.3) | 130(52.0) | 0.098 |  | 106(51.5) | 102(49.5) | 0.039 |
| Coronary heart disease | 97(5.1) | 68(27.2) | 0.163 |  | 44(21.4) | 41(19.9) | 0.036 |
| Diabetes | 239(12.6) | 54(21.6) | 0.126 |  | 41(19.9) | 41(19.9) | <0.001 |
| Cerebrovascular diseases | 43(2.3) | 27(10.8) | 0.164 |  | 13(6.3) | 16(7.8) | 0.057 |
| ACEI/ARB | 106(5.6) | 38(15.2) | 0.152 |  | 22(10.7) | 33(16.0) | 0.031 |
| Glucocorticoid therapy | 173(9.1) | 24(9.6) | 0.305 |  | 21(10.2) | 16(7.8) | 0.085 |
| Neutrophil count, ×10^9^/L | 3.28(2.53, 4.39) | 3.49(2.60, 4.72) | 0.220 |  | 3.29(2.48, 4.21) | 3.43(2.59, 4.65) | 0.006 |
| D-dimer, µg/mL | 0.45(0.23, 1.26) | 0.62(0.30, 1.58) | 0.142 |  | 0.47(0.31, 1.28) | 0.47(0.32, 1.10) | 0.092 |
| Total cholesterol, mmol/L | 4.23(3.62, 4.81) | 4.16(3.45, 5.36) | 0.056 |  | 4.15(3.48, 1.98) | 4.22(3.50, 5.15) | 0.126 |
| Triglyceride, mmol/L | 1.17(0.79, 1.70) | 1.39(0.87, 2.10) | 0.107 |  | 0.68(0.45, 1.00) | 1.37(0.89, 1.91) | 0.493 |
| LDL-C mmol/L | 2.54±0.70 | 2.61±0.95 | 0.010 |  | 2.41(1.87, 2.92) | 2.50(1.98, 3.11) | 0.171 |
| Procalcitonin, ng/mL | 0.04(0.03, 0.06) | 0.04(0.03, 0.07) | 0.071 |  | 0.04(0.03, 0.06) | 0.04(0.03, 0.06) | 0.133 |
| Creatine kinase–MB, ng/Ml | 1.16(0.80, 1.94) | 1.36(0.98, 2.04) | 0.104 |  | 1.19(1.19,1.21 ) | 1.19(1.19,1.49) | 0.067 |
| hs-TnI, ng/mL | 0.010(0.010, 0.011) | 0.010(0.010, 0.013) | 0.176 |  | 0.010(0.010,0.013) | 0.010(0.010,0.012) | 0.099 |
| BNP, pg/mL | 0.01(0.01, 44.64) | 14.37(0.010,83.24) | 0.055 |  | 10(10, 21.96) | 10(10, 22.16) | 0.120 |

Values are median (interquartile range), mean ± standard deviation or n (%). ^a^ Hospital indicates Leishenshan Hospital/Zhongnan Hospital of Wuhan University; ACEI=angiotensin-converting enzyme inhibitor; ARB=angiotensin receptor blocker; LDL-C=low density lipoprotein cholesterol; hs-TnI=high-sensitivity troponin I; BNP=brain natriuretic peptide.
