# Supplementary material for: Association of Statin Use With the In-Hospital Outcomes of 2019-Coronavirus Disease Patients: A Retrospective Study
Source: Front Med (Lausanne). 2020 Nov 17;7:584870. doi: 10.3389/fmed.2020.584870 (PMC7717990; doi:10.3389/fmed.2020.584870)
Supplement: Supplementary file 2 [file Table_2.docx]

**Supplemental table 2** Sensitivity analysis

|  | Total | Non-Statin | Statin | *P*-value | HR | 95%CI |
| --- | --- | --- | --- | --- | --- | --- |
| Sensitivity analysis 1  (excluding diabetes) |  |  |  |  |  |  |
| Number of patients | 414 | 207 | 207 |  |  |  |
| Outcomes-count (%) |  |  |  |  |  |  |
| Death | 13(3.1) | 10(4.8) | 3(1.4) | 0.039 | 0.256 | 0.070-0.931 |
| ARDS | 22(5.3) | 16(7.7) | 6(2.9) | 0.006 | 0.241 | 0.088-0.660 |
| ICU admission | 26(6.3) | 17(8.2) | 9(4.3) | 0.015 | 0.351 | 0.151-0.817 |
|  |  |  |  |  |  |  |
| Sensitivity analysis 2  (adding COPD) |  |  |  |  |  |  |
| Number of patients | 406 | 203 | 203 |  |  |  |
| Outcomes-count (%) |  |  |  |  |  |  |
| Death | 13(3.2) | 10(4.9) | 3(1.5) | 0.038 | 0.254 | 0.070-0.925 |
| ARDS | 22(5.4) | 16(7.9) | 6(3.0) | 0.006 | 0.240 | 0.088-0.659 |
| ICU admission | 26(6.4) | 17(8.4) | 9(4.4) | 0.015 | 0.351 | 0.151-0.816 |

Values are n (%). P-value was acquired by using the COX model. COVID-19=coronavirus disease 2019; COPD=chronic obstructive pulmonary disease; ARDS=acute respiratory distress syndrome; ICU=intensive care unit.
